# Next-day discharge after transcatheter aortic valve replacement in a Dutch hospital

**DOI:** 10.1007/s12471-025-01986-9

**Published:** 2025-09-04

**Authors:** Diekje R. Schouten, Josianne H. Heuver, Wendy Stouten-Gresnigt, Paulien Weijers, Esther van der Perk, Michiel Soullié, Joyce Peper, Benno J. M. W. Rensing, Jurriën M. ten Berg, Uday Sonker, Martin J. Swaans, Leo Timmers

**Affiliations:** 1 Department of Cardiology, St. Antonius Hospital, Nieuwegein, The Netherlands; 2 Department of Cardiothoracic Surgery, St. Antonius Hospital, Nieuwegein, The Netherlands

**Keywords:** TAVR, Patient discharge, Aortic stenosis, Length of stay, TAVI

## Abstract

**Background:**

In recent years, hospital stays after transcatheter aortic valve replacement (TAVR) have shortened. Previous studies have shown that next-day discharge (NDD) is feasible without compromising patient safety, but data from the Dutch hospital setting are lacking. To assess the real-world effect of a NDD policy after TAVR.

**Methods:**

A next-day discharge policy was introduced in 2022 at St. Antonius Hospital Nieuwegein, the Netherlands. We included elective TAVR patients between August 2022 and August 2024, excluding those with pre-existing hospitalisation, transapical access, or intraprocedural mortality.

**Results:**

Among 460 patients (mean age 80.1 ± 6.2 years, 40.9% female, and a median Edmonton Frail score of 3.0 [1.0–4.0]), the majority underwent transfemoral TAVR (99.1%), under local anaesthesia (97.0%), using self-expanding valves (78.3%). Patients in the NDD group were more often male, less frail, and less likely to have right bundle branch block before TAVR compared to delayed discharge (DD) patients. NDD was feasible in 269 patients (58.5%) with a low number of post-discharge complications at 30 days: 1.9% permanent pacemaker implantation and 2.2% minor vascular complications. There were no cases of mortality, major vascular complications, or in-hospital stroke. Main reasons for DD were conduction disorders, access site complications, and stroke, which contributed to a higher incidence of complications in the DD group (18.3% permanent pacemaker implantation, *p* < 0.001, 3.1% stroke, *p* = 0.004, 1.6% major vascular complication, overall *p*-value 0.02).

**Conclusion:**

After implementing an NDD policy, 58.5% of patients were eligible for NDD after TAVR with a very low post-discharge complication rate.

## Introduction

**Fig. 1:**
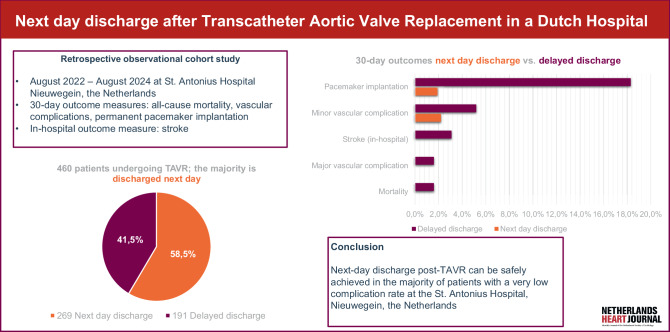
Infographic

Transcatheter aortic valve replacement (TAVR) is an established treatment strategy for patients with increased surgical risk and elderly patients with symptomatic severe aortic stenosis. One of the benefits of TAVR versus surgical aortic valve replacement is faster post-procedural recovery [1].

Over the past few years, the length of stay post-TAVR has shortened [2, 3]. Patients are increasingly discharged within 24–48 h post-TAVR. Some have even advocated TAVR as an outpatient procedure for selected patients [4, 5]. A recently published meta-analysis shows that next-day discharge (NDD) after TAVR is feasible without compromising patients’ safety. NDD does not affect mortality or readmission rates in selected patients deemed as being at low risk for complications [6]. However, no data are available from hospitals in the Netherlands. The efficient use of resources is increasingly important in the Netherlands due to a shortage of healthcare professionals, demographic aging, and the high prevalence of aortic stenosis in the elderly. TAVR is a minimally invasive intervention that reduces healthcare resource utilization compared to surgical aortic valve replacement [7, 8]. With the introduction of NDD after TAVR, these benefits are expected to be further reinforced (Fig. 1).

The St. Antonius Hospital in Nieuwegein, the Netherlands, implemented NDD post-TAVR in August 2022. The objective of this article is to assess the real-world effect of a next-day discharge policy after TAVR on feasibility, discharge timing, and short-term outcomes in this setting.

## Methods

### Study design

This study is a single-centre, retrospective, observational, cohort study. Data from August 2022 to August 2024 were retrieved from the electronic patient files by data analysts of the hospital and the researchers of this study.

### Study population

The study population includes all elective TAVR patients who were admitted to the St Antonius Hospital, the Netherlands, for TAVR. The hospital’s multidisciplinary heart team determined the TAVR indication in accordance with the Dutch guidelines for TAVR indications [9]. The access site, valve type, and valve size were determined during the heart team meeting.

Patients were excluded from analyses if they were hospitalised prior to TAVR, underwent a trans-apical procedure, or died during the procedure.

### Discharge policy

NDD was the intended strategy for all patients undergoing TAVR. The final timing of discharge was left to the discretion of the treating physicians, based on the patients’ clinical status post-TAVR and following the ESC guidelines for the management of valvular heart disease and cardiac pacing and resynchronisation therapy (2021) [10, 11].

### Patient characteristics and outcomes

Characteristics and outcomes of next-day discharge patients were compared to those discharged on day two or later post-TAVR. First, baseline characteristics were collected. At baseline, frailty was quantified using the Edmonton Frail Scale (EFS). Secondly, procedural and post-procedural variables were evaluated. Baseline ECG variables were assessed using the last ECG prior to TAVR, and post-procedural ECG variables were assessed using the first ECG obtained after TAVR.

The following 30-day outcomes were measured: all-cause mortality, vascular complications, and permanent pacemaker implantation (PPI). Stroke post-TAVR was measured as an in-hospital outcome. The vascular complications were divided into major and minor complications following the definitions of the Netherlands Heart Registration (NHR) [12]. Thirty-day outcomes were extracted from the electronic patient files of St. Antonius Hospital or requested from referring hospitals when applicable.

### Statistical analysis

The data were presented descriptively. Means with standard deviation (sd), medians with interquartile range (IQR), or absolute values with percentages were used. Comparisons between the NDD and delayed discharge (DD) group were made using the chi-square or Fisher’s exact test for categorical variables. For continuous variables, a t-test or Mann-Whitney U test was used. A *p*-value lower than 0.05 was considered statistically significant. Statistical analyses were performed using R Studio version 4.4.0.

## Results

### Study population

**Fig. 2:**
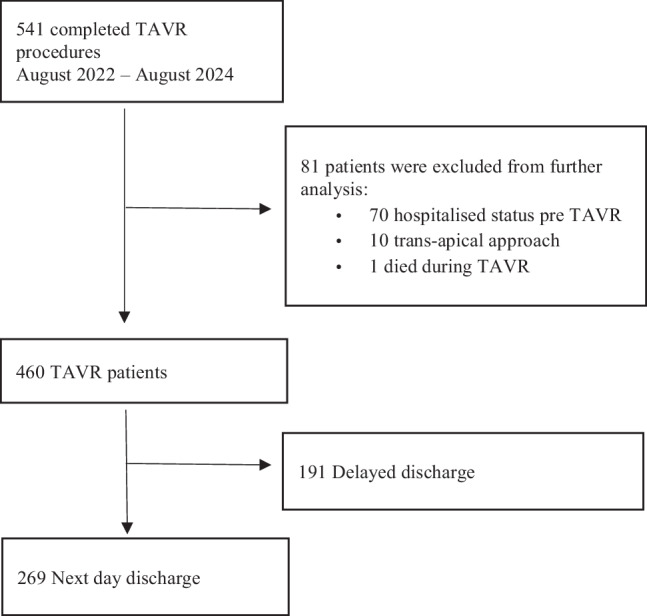
Flowchart study patients

Between August 2022 and August 2024, 541 TAVR procedures were performed. Eighty-one patients were excluded due to pre-TAVR hospitalisation, trans-apical approach, or intra-procedural death (Fig. 2). Four hundred and sixty patients are included in this study with a mean age of 80.1 years (SD: 6.2), 40.9% female, and a median EFS score of 3.0 (IQR 1.0–4.0). The majority of procedures were performed using the transfemoral approach (99.1%) under local anaesthesia (97.0%), using self-expanding valves (78.3%). No difference was found in the labeled valve size between both groups (data not shown).

Of all patients, 269 (58.5%) were discharged the next day post-TAVR, whereas 191 (41.5%) patients had a prolonged hospital stay. The median length of stay post-TAVR of patients with DD was 2.0 days (IQR 2.0–5.0). Additional baseline characteristics are shown in Tab. 1.

**Table 1:** Baseline characteristics

Variable	Next day discharge *n* = 269	Delayed discharge *n* = 191	Total *n* = 460	*p*-value
**Baseline**				
Age (years)	80.0 ± 6.4	80.3 ± 5.9	80.1 ± 6.2	0.61
Female	98 (36.4%)	90 (47.1%)	188 (40.9%)	0.03
BMI (kg/m^2^)	26.4 ± 4.3	26.4 ± 4.4	26.4 ± 4.4	0.99
Edmonton Frail Scale	2.0 [1.0–4.0]	3.0 [2.0–5.0]	3.0 [1.0–4.0]	0.01
Prior pacemaker	32 (11.9%)	14 (7.3%)	46 (10.0%)	0.12
Previous stroke	10 (3.7%)	10 (5.2%)	20 (4.3%)	0.49
*Diabetes Mellitus*				0.87
NIDDM	45 (16.7%)	32 (16.8%)	77 (19.7%)	
IDDM	19 (7.1%)	16 (8.4%)	35 (7.6%)	
*eGFR (ml/min)*				0.87
≥ 60	150 (56.0%)	111 (58.4%)	261 (57.0%)	
30–60	106 (39.6%)	71 (37.4%)	177 (38.6%)	
< 30	12 (4.5%)	8 (4.2%)	20 (4.4%)	
*NYHA*				0.42
I–II	42 (15.8%)	25 (13.1%)	67 (14.7%)	
III–IV	224 (84.2%)	166 (86.9%)	390 (85.3%)	
*LVEF*				0.63
< 30%	15 (5.6%)	13 (6.8%)	28 (6.1%)	
30–50%	70 (26.0%)	43 (22.5%)	113 (24.6%)	
≥ 50%	184 (68.4%)	135 (70.7%)	139 (69.3%)	
*ECG*				
AF pre	77 (28.6%)	64 (33.5%)	141 (30.7%)	0.30
1st degree AV block	41 (15.2%)	40 (20.9%)	81 (17.6%)	0.14
LBBB pre	31 (11.5%)	22 (11.5%)	53 (11.5%)	1.00
RBBB pre	11 (4.1%)	25 (13.1%)	36 (7.8%)	< 0.001

Values are shown as: mean ± standard deviation, median [interquartile range] or numbers with percentages

*BMI* Body Mass Index, *NIDDM* Non-insulin-dependent diabetes mellitus, *IDDM* insulin-dependent diabetes mellitus, *eGFR* estimated glomerular filtration rate, *NYHA* New York Heart Association classification, *LVEF* Left ventricular ejection fraction, *AF* Atrial fibrillation, *AV* Atrioventricular, *LBBB* left bundle branch block, *RBBB* right bundle branch block

At baseline, both groups did not differ significantly except for three variables. First, there are significantly more female patients in the DD group compared to the NDD group (47.1% vs 36.4% respectively, *p*-value 0.03). Second, the EFS is significantly higher (*p* = 0.01) in the DD group. At last, there are significantly more patients with a right bundle branch block (RBBB) in the DD group (*p* < 0.001). See Tab. 1.

There are also some significant differences between both groups in the procedural and post-procedural variables. There were significantly more patients in the DD group who experienced a peri-procedural 3rd degree AV block, compared to the NDD group (18.8% vs 1.9% respectively, *p* < 0.001). Additionally, the procedural duration was longer in the DD group (99 min with IQR 88–115) compared with the NDD group (90 min with IQR 81–105; *p* < 0.001). Post-procedural, significantly more patients in the DD group developed a 3rd degree AV block (*p* < 0.001), left bundle branch block (LBBB) (*p* = 0.04), or right bundle branch block (RBBB) (*p* < 0.001). For all procedural and post-procedural variables, see Tab. 2.

**Table 2:** Procedural- and post-procedural variables

Variable	Next day discharge *n* = 269	Delayed discharge *n* = 191	Total *n* = 460	*p*-value
**Procedural**				
*ECG*				
AV block				
3rd degree	5 (1.9%)	36 (18.8%)	41 (8.9%)	< 0.001
*Access route*				0.17
Femoral left/right	268 (99.6%)	188 (98.4%)	456 (99.1%)	
Axillary	1 (0.4%)	3 (1.6%)	4 (0.9%)	
General anaesthesia	7 (2.6%)	7 (3.7%)	14 (3.0%)	0.59
*Valve*				0.60
Evolut pro +/FX	125 (46.5%)	101 (52.9%)	226 (49.1%)	
Acurate Neo 2	83 (30.9%)	51 (26.7%)	134 (29.1%)	
Sapien 3 (Ultra)	43 (16.0%)	28 (14.7%)	71 (15.4%)	
Myval	18 (6.7%)	11 (5.8%)	29 (6.3%)	
Procedural duration in minutes	90 [81–105]	99 [88–115]	94 [83–109]	< 0.001
**Post procedural**				
*ECG*				
AF post	88 (32.7%)	71 (37.2%)	159 (34.5%)	0.37
AV block post				
1st degree	63 (23.4%)	48 (25.1)	111 (24.1%)	0.74
New 1st degree	22 (8.2%)	16 (8.4%)	38 (8.3%)	1.00
2nd degree Mobitz I	0 (0.0%)	0 (0.0%)	0 (0.0%)	
2nd degree Mobitz II	0 (0.0%)	2 (1.0%)	2 (0.4%)	0.17
New 2nd degree Mobitz II	0 (0.0%)	2 (1.0%)	2 (0.4%)	0.17
3rd degree	0 (0.0%)	22 (11.5%)	22 (4.8%)	< 0.001
LBBB post	90 (33.5%)	82 (42.9%)	172 (37.4%)	0.04
RBBB post	13 (4.8%)	30 (15.7%)	43 (9.3%)	< 0.001

Values are shown as: mean ± standard deviation, median [interquartile range] or numbers with percentages

*AF* Atrial fibrillation, *AV* Atrioventricular, *LBBB* left bundle branch block, *RBBB* right bundle branch block

### Outcomes

Among all TAVR procedures, 108 patients (23.5%) experienced a complication within 30 days after TAVR. In the research population, the 30-day mortality rate was 0.7%, the PPI rate was 8.7%, the rate of in-hospital stroke was 1.3% and the rates of vascular complications were 0.7 and 3.5% for major and minor complications, respectively (Tab. 3).

**Table 3:** Outcomes

	Next day discharge *n* = 269	Delayed discharge *n* = 191	Total *n* = 460	*p*-value
Mortality 30 days	0 (0.0%)	3 (1.6%)	3 (0.7%)	0.07
Permanent pacemaker implantation 30 days	5 (1.9%)	35 (18.3%)	40 (8.7%)	< 0.001
Stroke in-hospital	0 (0.0%)	6 (3.1%)	6 (1.3%)	0.004
*Vascular complications 30 days*				0.02
Major	0 (0.0%)	3 (1.6%)	3 (0.7%)	
Minor	6 (2.2%)	10 (5.2%)	16 (3.5%)	

Values are shown as numbers with percentages

### Mortality

In the NDD group, there were no cases of 30-day mortality, whereas three patients (1.6%) died in the DD group. Two of these patients died after discharge, 7 and 9 days post-TAVR. The cause of death was documented only for one of these two patients (acute myocardial infarction and cardiogenic shock). The third patient died in hospital fourteen days post-TAVR, due to respiratory insufficiency as a consequence of COVID-19 infection.

### Permanent pacemaker implantation

Five (1.9%) patients in the NDD group received a permanent pacemaker. All pacemaker implants occurred post-discharge. The timing of readmission varied between one and seven days post-discharge. Four patients showed a 3rd degree AV-block at readmission. One patient had new AF with slow ventricular response. Two of the five patients already had a first-degree AV block, and 1 patient had an RBBB pre-TAVR. These patients did not show new post-procedure conductance disorders. Two patients showed a new LBBB or RBBB post-TAVR. Of the 35 patients in the DD group who required a PPI, this indication accounted for DD in 33 cases (94.3%). Eighteen out of these 35 patients who required a PPI (51.4%) in the DD group showed a 3rd degree AV block during the procedure. In the remaining patients, the block occurred later during hospitalisation.

### Stroke and vascular complications

None of the patients in the NDD group had a stroke during the hospital stay post-TAVR. Additionally, no major vascular complications occurred in the NDD group. Minor vascular complications appeared in 2.2% of the patients in the NDD group. The rates of stroke and vascular complications were higher in the DD group and were also a reason for prolonged hospital stay. For the complication rates of both groups and *p*-values, see Tab. 3.

## Discussion

Previous studies identified NDD following TAVR as a safe strategy [6, 13–15]. As no Dutch data on NDD after TAVR exist from any hospital in the Netherlands, we evaluated its real-world effect on feasibility, timing, and short-term outcomes.

This study found low post-discharge complication and mortality rates in the NDD group. NDD was achieved in 269 (58.5%) patients. NDD rates vary across studies. Some report higher rates (72% [13], 80% [14]), but excluded patients with pre-existing RBBB, prolonged PR interval, AV block [13], or inadequate social support or mental stability [14]. In this study, NDD was the intended strategy for all TAVR patients, which may have contributed to a lower NDD rate.

The study population differed in three baseline variables. The NDD group included significantly fewer females compared to the DD group (36.4 and 47.1% respectively). Previous studies confirm that female sex is a negative predictor of NDD [13], as females often experience more bleeding and more vascular complications post-TAVR [16]. However, no sex-based differences in vascular complications were observed in this study.

Patients in the NDD group were less frail, with a median EFS score of 2.0 compared to 3.0 in the DD group. This aligns with findings by Holierook et al., which linked higher frailty scores to prolonged hospital stay [17]. Unlike other NDD safety studies [13, 14], we did not preselect based on social support, which is reflected in the EFS score and may explain group differences.

More patients in the DD group (13.1% versus 4.1% respectively) had a pre-existing RBBB. RBBB is a well-known predictor for the need of a PPI after TAVR [18], which may have caused a longer hospital stay in the DD group.

In this study, no cases of 30-day mortality were found in the NDD group. The overall 30-day mortality rate in this study was 0.7%, which is comparable to the findings of Eaves et al. [13]. Both studies reported no cases of mortality in the NDD group and found no significant difference between the two groups.

The incidence of PPI within 30 days post-TAVR was low in the NDD group (1.9%). PPI rates with NDD after TAVR ranged from 0 to 15.5% in the meta-analysis of Gupta et al. [6]. The occurrence of conduction disorders during or after TAVR often requires prolonged ECG monitoring or sometimes PPI, reflected in a higher rate of PPI in the DD group (18.3%), *p* < 0.001. Similar trends were reported in other studies [6, 13, 19].

Patients were readmitted with complaints of bradycardia, dizziness, and hypotension, most commonly due to 3rd degree AV block. All patients in the NDD group who received a permanent pacemaker were readmitted to the hospital between one and seven days post-discharge. Patients were discharged following the ESC guidelines on cardiac pacing and resynchronisation therapy 2021 [10], except for one patient. This patient showed a new LBBB with a QRS duration exceeding 150 ms after TAVR and, therefore, should have been monitored longer. However, the patient was readmitted with symptoms of bradycardia seven days after discharge, suggesting that 48 h of prolonged monitoring would not necessarily have altered the outcome.

Additionally, the rate of vascular complications was low in the NDD group. Respectively, 0.0 and 2.2% major and minor vascular complications occurred in the NDD group. The rates were higher in the DD group with 1.6 and 5.2% major- and minor vascular complications, respectively (*p* = 0.02). Especially major, but also minor vascular complications could be a reason for additional intervention and prolonged hospital stay. Two studies reported similar findings regarding differences in vascular complications between the groups [6, 13]. In contrast, the study of Ordoñez et al. found no difference in major vascular complications between both groups (*p* = 0.32) [19], and Eaves et al. found 1.5% major vascular complications in the NDD group [13].

In the NDD group, there were no patients with in-hospital stroke, whereas six patients (3.1%) had a stroke in the DD group (*p* = 0.004). Other studies found no significant difference in stroke. However, this outcome was measured at 30 days instead of in-hospital [6, 13, 19].

In summary, this study’s findings align with previous research. The low post-discharge complication rates in the NDD group in this study are consistent with expectations, as complications such as PPI, vascular complications, and stroke require prolonged observation or additional interventions during the post-TAVR hospital stay.

In our opinion, these findings likely apply to other Dutch TAVR-implanting hospitals, as both international and current data support the feasibility and safety of NDD. The results of this study did not prompt any changes to the discharge protocol.

The main limitation of this study is its single-centre, retrospective design. Second, bias may have been introduced because patients were selected for NDD by different physicians. However, all physicians followed the European guidelines and the aim of this study was to assess the practices in a real-life Dutch hospital setting.

Further research should explore factors contributing to prolonged hospital stay, including the timing of complication onset. Understanding the reasons behind prolonged hospitalisation could further optimise post-TAVR discharge protocols. Home monitoring to detect any arrhythmias and conduction disturbances could be explored.

## Conclusion

After implementing an NDD policy, 58.5% of the patients could be discharged the day after TAVR with very low post-discharge complication rates in a Dutch hospital setting. These findings are comparable to other international studies on NDD. Healthcare has been organised more efficiently since NDD was implemented. As the number of patients discharged the day after TAVR increases, healthcare consumption decreases, and costs can be minimised.
